# Bibliographical

**Published:** 1883-02

**Authors:** 


### Bibliographical.

*The Treatment of Diseases by the Hypodermatic Method: A Manual of Hypodermatic Medicatum.* Fourth Edition. By Robert Bartholow, M.A., M.D., LL.D. Philadelphia: Lippincott & Co. 1882.

The fourth edition of Prof. Bartholow's work forms a volume of over three hundred and fifty pages, showing how important and extended is this comparatively new method of medication.

Each of the thirty-four drugs he names as fitted for administration by this method, is treated of in the same methodical manner; but the physiological effects, it seems to us, occupy too much space in proportion to their value, when compared with therapy.

It is by no means proven that the action of drugs is the same in disease as in health; indeed, it is often markedly different. But Dr. Bartholow, here as in his work on "Materia Medica," lays great stress upon this division of his book, more so than is necessary for the recognition of symptoms of a toxic dose, which, for the therapist, is alone of value in this regard. However clear and elaborate the expositions are—and they are unsurpassed in this regard in the present work—they often belong to the domain of physiology, pure and simple, and in the modern scientific works on the last-named subject, will be found therein treated much the same as we find them in the manual for Hypodermatic Medication.

Prof. Bartholow uses the word *hypordermatic* in place of hypodermic, and asks aid in its introduction. The former is the correct term, as philology teaches us.

*A Treatise on the Physiological and Therepautic Action of the Sulphate of Quinine.* By O. T. Manson, M.D. Philadelphia: Lippincott & Co 1882.

Quinine has been known but three score years, yet its uses have, in that time, been extended till it nearly stands on a par with opium. In Doctor Manson's interesting little work, are given the history and modes of employment of the sulphate of quinine, in a concise, well-arranged and thorough manner. He has left no authority unquoted.

In yellow fever, our author seems to incline to the general belief, that little is to be expected from quinine. But in cerebro-spinal meningitis, he states (p. 150) that he has "obtained most satisfactory results." He regulated its administration by "the use of the thermometer;" if so, we do not see how he gave numerous "large doses," for cerebro-spinal meningitis is a disease where high temperature is uncommon, and where a fever of 102 or 103 degrees is not looked on as the element of danger. It is rather the effect of the blood-poison that we fear, and the pressure effects of an exudation about the base of the brain. Quinine is not generally looked upon as beneficial in cerebro-spinal meningitis.

Since the adoption of quinine in his treatment of croup, Dr. Manson has "not lost a case." He is fortunate.

The book, in its "get up," has only one fault—the absence of either index or table of contents.

*Caries and Necrosis of the Maxillary Bones.* By Truman W. Brophy, M. D., D. D. S. A concise and clear statement of the etiology, pathology, diagnosis and treatment of these lesions, with a history of illustrative cases. Dr. Brophy has abundant opportunities for the study of the subject through his professorship in Rush Medical College, and this paper is the first result of his observations, to be supplemented, we hope, by further reports of a yet more extended experience.

*The Minute Anatomy of the Teeth in the Light of the Bioplasson Theory.* By Carl Heitzman, M. D., and *The Minute Anatomy, Physiology and Therapeutics of the Dental Pulp.* By C. F. W. Bodecker, D. D. S., M. D. S.

We are in receipt of a pamphlet containing reprints of these notable contributions to our literature, and although both the papers have been before the profession for some time, their present reissue demands some notice.

The theory of the reticulated structure of the living matter in tissues, as announced by Carl Heitzman, has been bitterly assailed in many quarters as inconsistent with known facts. We do not propose to defend it, or even to announce an adherence to the doctrine. But that protoplasm is structureless, or that the protoplasmic elements of tissues and organs are isolated, disconnected, disassociated, is not in harmony with our ideas of function. What may be the nature of that interdependence is as yet a disputed question, and every reflecting anatomist will welcome the results of any exhaustive research which promises to throw additional light upon the vexed subject.

But some of Dr. Heitzman's conclusions do not seem to conclude. They are too vague and obscure for the comprehension of the average reader. Any statement of a scientific fact should be so clearly and concisely expressed that the purport may be apparent at once. If any reader of the pamphlet under consideration can grasp the meaning of its explanation of dental caries, he must have been under the personal instruction of its writer, or be blessed with an intuition that smiles at subtilties.

Dr. Bodecker has carried the investigation of dental tissues beyond the uttermost point reached by any of his predecessors. He has added to the sum of professional understanding a positive knowledge of many hitherto disputed questions. He has unmistakably demonstrated facts which were previously only surmised. There is probably no man now living who has a clearer and more intimate knowledge of the structure of the dental pulp than has Dr. Bodecker. When he speaks, therefore, he does it positively, and as one who knows whereof he affirms, and he is accepted as an authority, for however men may dissent from some of his conclusions they cannot impeach his knowledge of facts. The monograph under consideration is worthy the most careful attention of every thinking dentist. Nay, more: he who has not accorded to it a careful study is unqualified to form an intelligent opinion of the things concerning which the paper treats.

The *Medical Record* begins the present year with enlarged pages, and paper of finer quality. It greatly resembles the new weekly—but old publication—the *New York Medical Journal.* Both, we think, though now capable of containing more reading matter, are not of so convenient a form as of old.

The *Planet* is an aspirant for medical journalistic honors. It is "a very little one," but, as the French proverb goes, all comes to him who can wait.

The *Medical Age* is a consolidation of the *Michigan Medical News* and the *Detroit Clinic.* Dr. Mulheron is the editor.

The first number of the *Medical and Surgical Herald* comes from Missouri, as the organ of the Joplin College of Physicians and Surgeons.

*L' Union Medicale du Canada* enters the new year, with Drs. Lamarche and Desrosiers as editors, and with such an arrangement of type that ten pages more of matter are given monthly.

*Pocket Therapeutics and Dose Book.* By Morse Stewart, Jr., B.A., M.D. Third edition. Detroit: 1882.

A very handy and neat little volume, showing great care in its compilation.

*Bulletin de l'Academie Royale de Medicine de Belgique.* Bruxelles: December, 1882.

The last instalment of the *Bulletin* for 1882, completes a yearly volume of 1,116 pages. In this, the last number, are several most interesting discussions and papers concerning the so-called "vaccinating and anti-vaccinating schools," the treatment of "diabetes mellitus with permanganate of potash," and the account of some unique surgical operations.

Pamphlets Received.—*Contribution to Surgical Gynecology.* By Edward W. Jenks, M.D., LL.D. Chicago: 1882. *Modified Listerism in Ovariotomy, with a Report of Five Recent Operations.* By Edward W. Jenks, M.D., LL.D. Chicago: 1882. *Bromide of Ethyl, the most perfect Anæsthetic for short painful surgical operations.* By Julian J. Chisholm, M.D., Professor in the University of Maryland. Baltimore: 1883. *Annual Address Delivered before the American Academy of Medicine*, by Traill Green, A.M., M.D., President of the Academy, at Philadelphia, October 26, 1882.

*Dental Chart.* At the annual meeting of the Dental Society of the State of New York, held in Albany in May, 1882, Dr. Frank Abbott presented a diagramatic representation of a vertical section through the centre of an incisor tooth and all the adjacent and investing tissues. It was upon a scale of such magnitude as to represent all the elements as they would be seen through a microscope magnifying some thousands of diameters, the chart being about seven feet in length All the minute histology of the tooth, with the environing tissues was faithfully represented in colors, and it was pronounced at the time the finest thing of the kind yet produced.

The State Dental Society took immediate action, voting that it should be published in some way for the benefit of the profession at large, and appointing a committee to act with Prof. Abbott in securing so desirable a result.

Through the courtesy of the S. S. White Dental Manufacturing Company, we are now in receipt of a fac simile of the original, printed in more colors than we care to enumerate, upon cardboard 14 x 20 inches in size, with full reference explanation upon the margin, forming altogether the most beautiful dental chart that we have yet seen. Suitably framed, it would not only form a rare ornament for the walls of any dental office, but it would prove invaluable as a guide to the study of the intimate structure of dental tissues.

